# “HEAL together”: a randomized, hybrid type 1 effectiveness-implementation trial protocol of a peer-delivered behavioral activation intervention to improve methadone treatment retention

**DOI:** 10.3389/fpubh.2025.1637846

**Published:** 2025-07-18

**Authors:** Jessica F. Magidson, Valerie D. Bradley, Jessica S. Anane, Mary B. Kleinman, Julia W. Felton, Abigail C. Hines, Rithika Baskar, Aaron D. Greenblatt, Dwayne Dean, Morgan S. Anvari, Heather Fitzsimons, Melanie E. Bennett, Annabelle M. Belcher

**Affiliations:** ^1^Department of Psychology, University of Maryland, College Park, College Park, MD, United States; ^2^Center for Substance Use, Addiction & Health Research (CESAR), University of Maryland-College Park, College Park, MD, United States; ^3^Henry Ford Health, Detroit, MI, United States; ^4^Department of Psychiatry, University of Maryland School of Medicine, Baltimore, MD, United States

**Keywords:** peer recovery specialists, behavioral activation, medication for opioid use disorder, retention, methadone, opioid use disorder

## Abstract

**Background:**

Although medications exist to effectively treat opioid use disorder (OUD), treatment retention is a pressing challenge. Peer recovery specialists (PRSs) may play an important role in OUD treatment retention, yet few evidence-based interventions to support OUD retention have been developed specifically for PRS delivery. Behavioral activation is a brief, reinforcement-based intervention with empirical support for improving depression and substance use outcomes, delivered typically by specialist mental health providers. Informed by key stakeholder feedback, our team adapted a behavioral activation and problem-solving intervention for PRS delivery (“*Peer Activate*”) to improve methadone treatment retention. Building on a successful open-label pilot trial demonstrating initial feasibility, acceptability, and preliminary effectiveness of *Peer Activate,* the current type 1 hybrid randomized controlled trial evaluates the effectiveness of *Peer Activate* compared to treatment as usual on six-month methadone retention (primary) and longer-term implementation outcomes.

**Methods:**

The trial is being conducted at a large methadone treatment program in Baltimore City, Maryland. We are enrolling 200 patients who recently initiated methadone treatment or are experiencing challenges with methadone adherence in a randomized 1:1 ratio to receive *Peer Activate* plus treatment as usual (PA + TAU) or TAU only. Additionally, we are recruiting 12 stakeholders to provide feedback on implementation and sustainability. *Peer Activate* consists of four core intervention sessions delivered by a PRS with relevant lived experience and training in the intervention. Sessions focus on problem-solving barriers to retention and behavioral activation—increasing value-driven, substance-free activities—and continued skill practice and relapse prevention. Assessments are administered at baseline, post-treatment (approximately 3 months), and 6 months. The primary effectiveness outcome is methadone retention over 6 months, measured using chart review. Implementation outcomes are defined based on Proctor’s model, including feasibility, acceptability, and fidelity of the intervention.

**Discussion:**

This trial will provide insight as to whether a PRS-delivered intervention may be effective and feasible for improving methadone treatment retention and other behavioral health outcomes. If findings are promising, *Peer Activate* may provide a platform on which to incorporate an evidence-based behavioral activation approach into PRS training nationally.

**Clinical trial registration:**

NCT05299515.

## 1 Introduction

Opioid use disorder (OUD) and opioid overdose deaths have increased profoundly in the past decade, exacerbated further by the COVID pandemic (1, 2). Between October 2023 and September 2024, approximately 87,000 people died of drug overdoses in the United States (3), and opioids are a factor in seven out of every ten drug overdose deaths (4). While there has been a recent decrease (3), overdose deaths still disproportionately affect certain groups, including low-income racial and ethnic minoritized groups, particularly Black/African-American individuals living with OUD (5). In Maryland, the setting for the current trial, Black men have experienced an almost fivefold increase in mortality due to drug overdoses between 2010 and 2020 (6). Baltimore City, in particular, experiences a staggering number of drug overdose deaths with a fatal overdose rate of 15 per 100,000 residents between August 2023 and July 2024—nearly five times that of the national rate (7).

Medication for OUD (MOUD) is the gold standard evidence-based treatment for OUD (8, 9) to reduce cravings, drug use, and prevent overdoses (10, 11). MOUD includes the medication options of methadone and buprenorphine, which are prescribed and administered depending on patient and provider needs and access. Methadone treatment is the oldest and currently most common treatment option, especially among low-income and racially minoritized patients (12). This trial focused on methadone retention for a few important reasons. First, methadone treatment typically requires daily dosing that is highly regulated and includes frequent, often daily, in-person observed dosing; yet, retention in methadone treatment is often less than 60% within six-months of treatment initiation (13, 14), and even lower among low-income, marginalized individuals (14–16). Retention is associated with many other indirect benefits, including a reduction in criminal convictions and improved physical health and quality of life (17, 18). It is imperative to develop and test new strategies to improve methadone treatment retention rates and focus on the barriers faced by groups that are most affected by OUD.

There is growing evidence that the inclusion of peer recovery specialists (PRSs) in substance use treatment programs can improve MOUD retention (19, 20). PRSs are trained and often certified individuals with their own lived experience of substance use and recovery. PRS-delivered interventions can offer a more informal and personalized interaction for patients to receive support navigating the healthcare system, build healthy relationships, and strengthen their support system (21). There is evidence that PRS-delivered interventions are high in feasibility and acceptability (22–24), as well as evidence of positive patient interactions and support of PRSs (23). Moreover, research has established that PRSs can deliver brief, behavioral evidence-based interventions (EBIs), including behavioral activation and contingency management, with high fidelity while incorporating their lived experience into the interventions (22, 25–27). This may especially be true when PRSs are involved in the intervention adaptation process and view the intervention components as in-line with the PRS role (26). Yet, few trials have evaluated more structured EBI delivered by PRSs to support MOUD retention. By incorporating their lived experience, a PRS may increase trust and thereby foster greater patient engagement in EBIs while also maintaining fidelity to the PRS role and the intervention.

Grounded in reinforcement theory, behavioral activation (BA) is a promising EBI with empirical support to improve treatment retention. BA targets positive reinforcement to enhance retention in substance use treatment (28) and prevent relapse (29–32) by promoting engagement in value-driven, substance-free activities that increase enjoyment and mastery. Additionally, when combined with problem-solving interventions, BA improves medication adherence (e.g., for HIV) among low-income, minoritized populations with substance use disorder (29, 33–35). From an implementation perspective, we and others have shown that BA is feasibly delivered by PRSs and other lay health workers and represents an EBI that is in line with the PRS role (34, 36, 37). Additionally, it is able to be implemented by non-specialists (38, 39), particularly in low- and middle-income countries (22). However, limited prior work has evaluated PRS delivery of BA in the US to improve OUD treatment outcomes.

Our team conducted extensive prior work, including several rounds of iterative qualitative research with patients, PRSs and stakeholders, to adapt BA for PRS delivery, specifically focusing on improving methadone treatment retention (23, 40). We then conducted an open-label pilot trial (*N* = 37) of this adapted *Peer Activate* PRS-delivered BA approach. This pilot demonstrated preliminary effectiveness in improving methadone treatment retention, as well as acceptability, feasibility, and fidelity (24). Based on our pilot study and prior research, BA delivered by a PRS appears to be an ideal EBI for improving methadone treatment retention. The present trial builds upon this formative work and successful pilot (24).

### 1.1 Trial objectives

This hybrid type 1 effectiveness-implementation randomized trial aims to evaluate the effectiveness and implementation of *Peer Activate* compared to treatment as usual (TAU) at a large methadone treatment center in Baltimore. The primary effectiveness outcome is methadone treatment retention over six months. Guided by Proctor’s model (41) of implementation, we are evaluating feasibility, acceptability, and fidelity using mixed methods.

## 2 Materials and methods

### 2.1 Setting and recruitment

The study is conducted at a large substance use treatment center in Baltimore City that is affiliated with a local academic medical center, based in a community setting. The program currently serves over 425 active patients. Participants for the trial (*N* = 200) are recruited from the methadone treatment program through various screening methods: reviewing medical records of recently enrolled patients who have consented to being contacted for research at intake, provider/staff referrals, and through setting up informational tables to engage with participants in the clinic waiting room.

### 2.2 Eligibility criteria

Inclusion criteria for study eligibility are: (1) ≥18 years of age; and (2) initiating treatment in the methadone treatment program in the last three months (and having been enrolled in methadone treatment for at least two weeks) or demonstrating challenges with methadone adherence within the last three months, defined as: (a) at least one missed methadone dose in the past three months; (b) at least one missing methadone take-home bottle at the time of bottle return; (c) negative urine toxicology results for methadone in the past three months; and/or (d) transition from extended methadone take-homes to daily dosing due to concerns regarding adherence. These criteria were developed based on physician and stakeholder feedback and piloted previously (24). Exclusion criteria include: (1) pregnant at study enrollment; (2) untreated or undertreated psychosis or mania that would interfere with study participation; and/or (3) inability to provide informed consent in English.

### 2.3 Informed consent and ethical considerations

Trained members of the research team conduct informed consent with participants. After reading the consent form or having it read to them, participants are asked three structured questions to verify their understanding of the study, and any questions are answered. With participant permission during informed consent, all intervention sessions are audio recorded for supervision purposes and PRS intervention fidelity monitoring. This is noted in the consent process, and participants can refuse audio recording at any time (refusal of audio recording does not preclude participants from continuing to be part of the study). As part of the informed consent process, participants are advised that they may decline to answer any questions. This provides participants with the assurance of confidentiality around sensitive personal information relating to substance use and mental health. All personnel working on the project receive training on participants’ rights to confidentiality, and all study personnel are appropriately trained in the ethical conduct of human subjects’ research.

As this work includes collection of data relating to illegal behavior and substance use, the National Institutes of Health has provided the study with a Certificate of Confidentiality. As part of the consent process, participants are informed that, under the Certificate of Confidentiality, researchers cannot release or use information, documents, or samples that may identify participants in any action or suit unless participants provide permission, including in federal, state, or local legal action.

The University of Maryland, College Park Institutional Review Board (IRB) reviewed and approved all study procedures, which were approved through an Interagency Agreement with the University of Maryland, Baltimore. All study procedures are overseen by the PI.

This project is monitored by a Data and Safety Monitoring Board (DSMB) that meets annually to oversee the trial. The DSMB members function free of the career and financial interests of its members and consists of four members with experience in conducting clinical intervention research for psychiatric disorders, expertise in clinical trial ethics, human subject protection issues working with underserved populations, and peer recovery and policy expertise, including lived experience. The DSMB is updated annually. Annual meetings take place in the form of either a remote review of the annual DSMB report or a conference call to review project progress, barriers and challenges to study progress, as well as adverse events. The DSMB is presented with major amendments to the study protocol, study drop-outs, and determines whether study procedures should be changed or the study should be halted for reasons related to the safety of study subjects.

### 2.4 Assessment schedule

**Figure 1:**
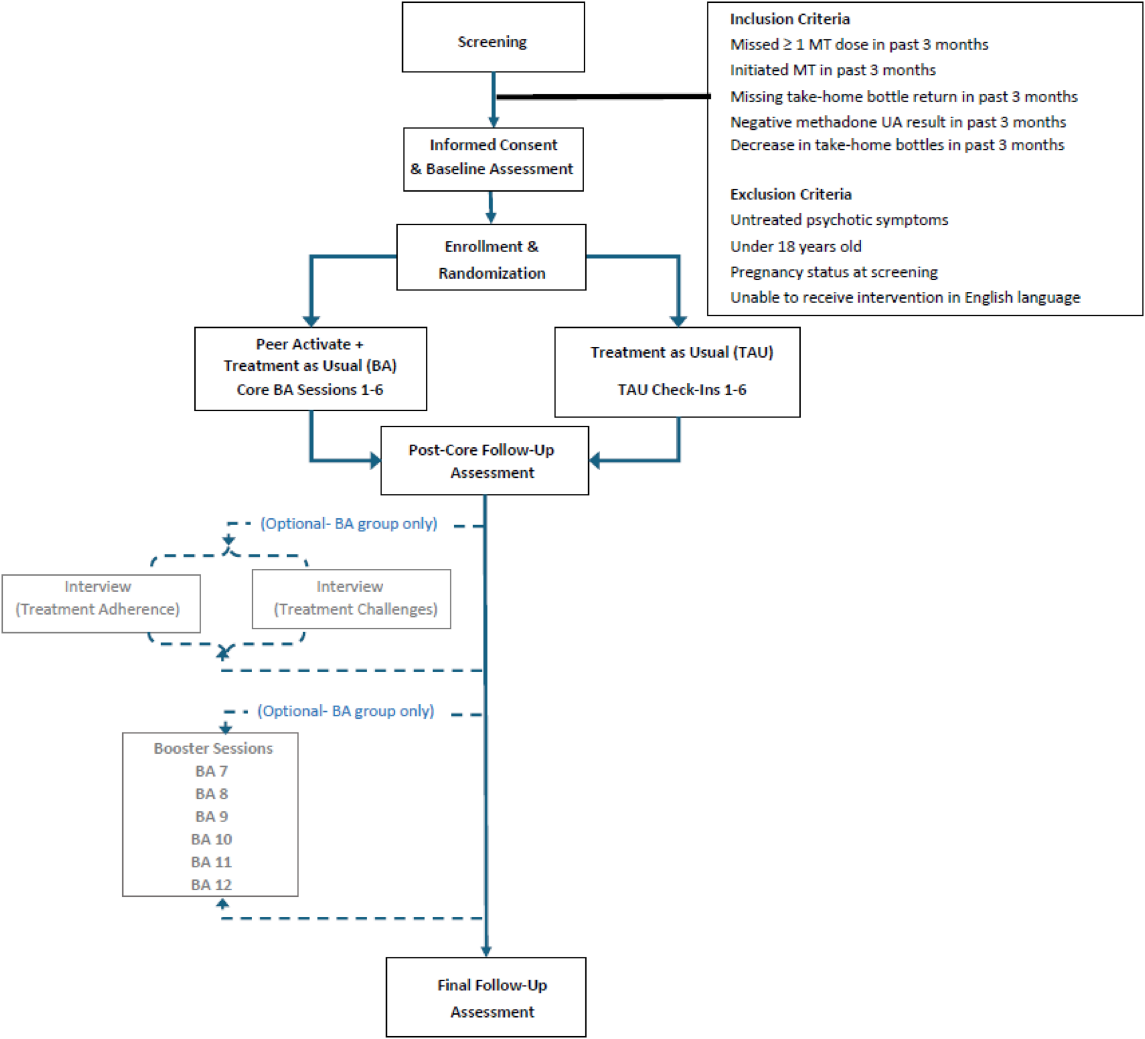
Study flow diagram — patient participants.

Participants first complete a baseline assessment, followed by assessments at approximately three- and six-months post baseline (see assessment measures in Figure 1). Both conditions (*Peer Activate* and TAU) have the same study assessment schedule. Participants are compensated $25 for each assessment. The study follows an intent-to-treat approach; participants can choose to continue study assessments without engaging in intervention sessions and/or withdraw at any time.

### 2.5 Baseline assessment

The baseline assessment includes a mix of self-report assessments of primary outcome variables at baseline (see Table 1) and other demographic and clinical characteristics. The baseline self-report assessments are administered by a research assistant using REDCap. Final eligibility is confirmed after conclusion of the baseline assessment. Patients who present with active psychotic or manic symptoms are further assessed using the Mini-International Neuropsychiatric Interview (MINI) (42); if symptoms are deemed to be active, untreated, and likely to interfere with study procedures, these participants are deemed ineligible and provided additional internal and external resources for treatment and other psychosocial support.

**Table 1:** Study outcomes measured at baseline, post-core follow-up, and final follow-up assessments.

Study outcomes and associated measures	Timeframes		
	Baseline (BL)	Post core follow-up (~6–12 weeks post-BL)	Final follow-up (~18–24 weeks post-BL)
Effectiveness outcomes (Primary)			
Methadone treatment retention - *Defined dichotomously as retention (yes/no) in methadone treatment* - Medical chart extraction		X	X
Methadone treatment persistence - *Calculated as the proportion retained on methadone treatment monthly (*i.e.*, at least one methadone dose for each 30 day period)* - Medical chart extraction		X	X
Implementation outcomes (Secondary)			
Intervention feasibility % patients who initiated intervention Qualitative interviews		X	
Intervention acceptability % patients enrolled who attended ≥75% core intervention sessions Qualitative interviews		X	
Intervention fidelity % of intervention components delivered as intended (20% of sessions, randomly selected)		X	
Other outcomes			
Effectiveness			
Changes in depressive symptoms			
PHQ-8	X	X	X
Changes in substance use			
Urinalysis Timeline Followback	X X	X X	

### 2.6 Randomization

Eligible participants are then randomized to either treatment as usual (TAU) or *Peer Activate* (PA + TAU), described below.

### 2.7 Treatment as usual (TAU)

TAU includes standard of care at the methadone treatment program, including a referral resource sheet, inclusive of general health centers, mental health providers, substance use support services, urgent care, domestic violence support, crisis hotlines, and other community services. Methadone treatment at the study site typically includes daily, observed methadone dosing 5 days per week with weekend take-home doses. Based on medical team determination, Saturday in-person dosing is also available and provided to patients who demonstrate the need for additional support (i.e., concern about the safety of medication storage in unstable housing settings, etc.). Depending on medication adherence (missed doses) and routine (approximately monthly) urine toxicology results, patients can receive up to 28 days of take-home doses of medication, provided at increasing intervals based on discussion across the treatment team (addiction medicine providers and addiction counselors). Patients who receive take-home bottles are required to return empty bottles at their next visit. Note, all data for this trial are being collected following COVID-19 modifications that eased federal regulations governing methadone dosing practices. Prior to these changes (issued in March 2020), patients were required to fulfill stringent criteria before considerations were given to decrease their observed dosing schedules and/or take-home eligibility (43, 44). Participants enrolled for this trial represent patients who have had variable experiences with pre- and post-COVID-19 pandemic methadone treatment dosing modifications. Clinic services also include wrap-around services such as individual and group counseling (which do not include BA or problem-solving therapy), primary care and infectious disease treatment, health home services, and access to peer recovery support at the site (i.e., general peer psychosocial support and service navigation). Thus, all participants in the trial have access to work with a peer in the traditional PRS role.

### 2.8 Peer-delivered BA intervention (“*Peer Activate*”)

The core components of the PRS-delivered intervention (“*Peer Activate*”) include: (1) BA adapted for substance use (30), iteratively adapted by our team to be feasible for PRS delivery and focused on methadone treatment retention (23, 40); and (2) problem-solving strategies to support retention in methadone treatment adapted from Life Steps for medication adherence (45). Sessions begin with a check-in on substance use and methadone treatment adherence, followed by problem-solving to address barriers to adherence, for instance transportation and housing, and scheduling substance-free, value-driven rewarding activities.

*Peer Activate* consists of six weekly sessions (approximately 30–45 min each). The first four sessions introduce key content, followed by two core review sessions, and six optional booster sessions to further reinforce skill practice. Booster sessions address relapse prevention, balancing activities, building social support, and planning for challenges (24). This format and approach were adapted based on stakeholder and participant feedback from the pilot study (24). PRSs share their lived experiences to reduce stigma, increase engagement, and motivate participants (46). A flipbook format is used to promote peer fidelity to the behavioral intervention components, with specific prompts to incorporate one’s own lived experiences into intervention delivery as we have done in other trials evaluating PRS-delivered behavioral interventions in international settings (31). Table 2 outlines the core components of the intervention (see also (46) for greater detail on the intervention and flipbook delivery in a case series demonstration).

**Table 2:** In-depth description of intervention components.

Intervention component	Description	Homework
Life steps	Aims to teach problem-solving strategies for medication adherence Work with participants to identify barriers to adherence that they experience (e.g., transportation, social support, etc.) Develop a plan and a back-up plan to overcome barriers Continuously check-in and refine strategies	Monitor barriers to adherence Utilize plans to overcome barriers
Psychoeducation/ behavioral activation rationale	Describe the behavioral cycle of substance use whereas urges lead to negative behaviors, which lead to negative emotions, and so forth Work with participant to identify that while we cannot control our urges, we can decide to engage in more positive, rewarding behaviors, which then lead to more positive emotions	
Behavior monitoring	Asks participants to monitor their daily activities, including specificity to activity details (with whom, where, etc.) and time frames Includes recording feelings and substance cravings on a scale of 1–10 Work with participant to differentiate what differences in mood/cravings look and feel like for them	Complete behavior monitoring homework sheet during the upcoming week
Life values	Describe what a value is Work with participant to identify their values across various life areas (relationships, mental/physical health, hobbies, career/education, spiritually, or other participant-identified areas)	
Activity scheduling	Identify activities that are rewarding, do not involve substances, and are in-line with participants pre-identified values As an example, chose one value and identify at least two activities for that value Work with participant to develop a plan for scheduling the identified activities into their upcoming week	Complete activity scheduling worksheet throughout the week, keeping track of activities and associated mood and craving levels

Sessions are held in-person at the methadone treatment center, with the option to complete some virtual study procedures (i.e., via phone, Zoom, or Google voice) when not feasible to meet in person for health/safety concerns, scheduling challenges, or other pertinent reasons. While all intervention sessions are scheduled, the PRS schedule is also flexible to meet patients on a walk-in basis to best support participants demonstrating ongoing challenges with treatment attendance and consistency in care. The PRSs document how and when sessions take place (method of communication, physical locations of PRS and participants, time of day) to further our understanding of how the PRS role can optimize participant engagement in treatment. PA + TAU participants receive $5 at each intervention session to support any additional travel costs.

### 2.9 PRS interventionists and training

Two study PRSs are trained and supervised in core peer recovery skills, knowledge, and abilities, are certified or working toward certification as PRSs in the State of Maryland and receive training in the study protocol and intervention procedures. Training is led by a PRS supervisor who was the primary PRS interventionist during the prior open-label pilot phase of this project (24). As an Internationally Certified Peer Recovery Specialist, a Maryland State Certified Peer Recovery Specialist, and a Registered Peer Supervisor through the Maryland Addictions and Behavioral Health Certification Board, the PRS supervisor has extensive experience in the training and certification of PRSs. The other PRS was recruited through hiring announcements and word of mouth, was a novice to the field of peer recovery who has lived experience in substance use and recovery. Training was conducted over approximately 1 month and includes a mix of didactic teaching, demonstrations, and role play of the peer role and *Peer Activate* intervention, with ongoing booster training and practice included in weekly supervision.

*Peer Activate* intervention training was guided by the intervention flipbook and reinforced through role-plays and shadowing. The PRS trainee shadowed the PRS supervisor in intervention sessions for up to 2 weeks before leading *Peer Activate* intervention sessions with the PRS supervisor present to observe for an additional 2 weeks. Once the PRS supervisor determined the PRS demonstrated competence, the PRS adopted an independent caseload under the supervision of the PRS supervisor. The PRS supervisor reviewed recorded practice sessions and guided the PRS through ongoing weekly clinical and PRS supervision as outlined below.

We did not restrict hiring to certified PRSs; rather, we provided support toward PRS certification as part of study procedures. Maryland Certified Peer Recovery Specialist (CPRS) certification is managed by Maryland Addictions and Behavioral Health Professional Certification Board and requires 46 hours of training, 500 work hours in peer recovery support, and 25 hours of documented supervision under a registered PRS supervisor (47). Maryland CPRS certification requires a core training that aligns knowledge, skills, and abilities in four subject domains: Advocacy, Mentoring & Education, Recovery & Wellness, and Ethical Responsibility (47). PRS training includes instruction on peer recovery skills, motivational interviewing, self-care and personal wellness planning, PRS role division and referral processes for clinical care (such as suicidal risk response and psychological support), ethics and boundary management, as well as disclosure as a recovery support tool. Once hired, the PRS also received supervised hours from a Registered Peer Supervisor toward PRS certification. The PRS supervisor provided support throughout the peer certification process, including sharing resources to acquire requisite trainings, preparing the peer for the examination, and supporting the PRS interventionist through the overall process. This support for general PRS training and certification was provided in addition to the study specific *Peer Activate* training.

### 2.10 PRS supervision

The approach to supervision includes both PRS supervision led by a PRS supervisor and clinical supervision led by a clinical psychologist. Weekly supervision sessions with a PRS supervisor include real-time case reviews of participant sessions where the PRS interventionist can raise questions and seek guidance, and the PRS supervisor can assess PRS performance in the intervention administration after listening to session recordings for fidelity monitoring. Based on these discussions, the PRS supervisor reviews relevant content and provides ongoing training to the PRS interventionist and supports resource brokering to ensure the PRS interventionist can refer participants to various community resources, as needed. PRS supervision sessions equally focus on knowledge-building and skills development with the goal of enhancing the understanding of systems of care, pathways for MOUD, the goals and agenda of MOUD care, person-centered support, and reducing barriers to receiving care. Additionally, PRS interventionists engage in twice weekly check-ins, ranging from 30- to 60 min in length, as required, to address real-time developments in training and delivery of the intervention, including regular monitoring of session fidelity as well as one’s own recovery and self-care. The PRS supervisor and PRS interventionists also consult on a weekly basis with clinical psychologists on the team to refer for higher level clinical needs and referrals, as well as to preserve role boundaries for PRSs and have sufficient support from licensed mental health clinicians. Clinical psychologists are also available to support ongoing booster training needs for BA and problem solving skills.

### 2.11 Follow-up assessments

Participants from both study groups (PA + TAU and TAU) complete follow-up assessments: post-core intervention sessions follow-up (PCFU) approximately six to 12 weeks post baseline and final follow-up (FFU) approximately 18–24 weeks post baseline. Follow up assessments are conducted by an independent blinded assessor who is trained on all study procedures but outside the core study team and blinded on randomization status. Measures administered are similar to the baseline assessment (see Table 1). Intervention implementation measures are completed during the PCFU assessment by an unblinded research team member and assess feasibility, acceptability, and fidelity among participants who received the *Peer Activate* intervention.

### 2.12 Effectiveness outcomes

#### 2.12.1 Primary outcomes

##### 2.12.1.1 Methadone treatment retention

The primary effectiveness outcome for this trial is methadone treatment retention, which is assessed over six months post baseline. Methadone treatment retention is examined through clinical records of appointment attendance and determined based on one or more methadone treatment-related visit(s) attended for each 30-day period during the six months following baseline. The primary outcome is retention assessed dichotomously (i.e., yes/no) at approximately six months post baseline. Participants who are referred to another internal treatment program or transferred to an external methadone treatment program are considered retained at six months based on clinic-verified records. To supplement the dichotomous measure of retention, we will also assess methadone treatment persistence, defined as the proportion retained on methadone treatment monthly over six months (i.e., at least one methadone dose for each 30-day period) post baseline, and days retained in treatment.

#### 2.12.2 Secondary outcomes

We will also assess methadone treatment retention and persistence at three months post baseline, as well as changes in substance use over six months using both urinalysis and timeline follow back methods and depressive symptoms using the PHQ-8. For implementation outcomes, we will assess adoption of the intervention qualitatively through focus groups with providers and other key stakeholders (*n* = 12) about intentions to implement the intervention following the trial and to inform future adaptations to *Peer Activate* and plans for adoption and sustainability. Focus groups will be led with a semi-structured guide using Proctor’s model to define adoption (41).

#### 2.12.3 Implementation outcomes

Implementation outcomes were defined based on Proctor’s model (41), including feasibility, acceptability, and fidelity, using mixed methods as detailed below.

##### 2.12.3.1 Feasibility and acceptability

Feasibility and acceptability are assessed by session attendance (both initiation of the intervention and attendance at core sessions, respectively, See Table 1) and using a validated quantitative measure of feasibility and acceptability, the Applied Mental Health Research Group implementation outcome assessment (48). This assessment has been used to evaluate the implementation of interventions delivered by peer and lay health workers internationally and was designed to be adaptable for use in different resource-constrained settings globally (34, 49). In addition to quantitative measures, we are collecting qualitative feedback from patients (interviews offered to *Peer Activate* participants demonstrating either high treatment adherence or challenges with treatment during study engagement; *n* = 40) about their experiences with the intervention with specific probes related to feasibility and acceptability based on Proctor’s definitions (41). Qualitative and quantitative measures of feasibility are assessed at the posttreatment follow-up assessment (approximately three months post-baseline) or in the event of early participant discontinuation of the study.

##### 2.12.3.2 Intervention fidelity

All *Peer Activate* sessions are audiotaped with patient permission as part of the consent process, and a randomly selected 20% are reviewed by a trained, independent rater, assessing both adherence and competence (50). The PRSs also self-report intervention adherence following each session. An adapted rating checklist for PRS adherence is utilized to determine whether the specific treatment components were delivered (both PRS report and independent ratings). Following recommendations for implementation science research (51), a “fidelity score” is calculated based on the proportion of key intervention components delivered as intended across sessions. Competence in the domain of general clinical skills (e.g., empathic listening, non-judgment) is rated using the ENhancing Assessment of Common Therapeutic Factors (ENACT) rating scale (52), a cross-cultural competency measure of skills of lay counselors, including PRSs specifically, in delivering a behavioral intervention. Prior research has seldom captured how to balance delivery of an evidence-based intervention, such as BA, with sharing one’s own identity as a PRS or adhering to the PRS role. The combination of these fidelity measures allows us to capture these unique elements of a PRS-delivered intervention (25).

### 2.13 Retention of study participants

As a strategy to support retention in a traditionally hard-to-reach population, both groups engage in approximately weekly contact (in-person or by phone) with the research team to continue to verify contact information to promote study retention. At these contact verifications, the research team asks brief questions about self-reported methadone treatment adherence and any changes to treatment enrollment (whether they are still enrolled in methadone treatment as well as any substance use treatment programs outside the study site clinic setting). Participants receive $5 compensation for completing each contact verification visit.

### 2.14 Power considerations and sample size calculations

Sample size estimates were calculated using PASS 16.0. Estimates for primary outcomes (retention and number of days in treatment) were calculated assuming: (1) 20% anticipated attrition in patient participation over six months, based on our preliminary work (24); (2) 2-tailed alpha = 0.05; and (3) a 1:1 enrollment ratio. Given prior findings suggesting the proportion of retention in the control group is 0.5 at 180 days, a sample size of 200 (100 patients per group) with an expected retention of 80% at six-month follow-up gives us 80% power to detect a 0.18 proportion difference in retention across conditions. While a binary outcome (retention/attrition) is used in our primary analyses, we can also examine a continuous measure of number of days in treatment. For these models, we would estimate 80% power to detect a small-to-medium effect size (*d* = 0.44).

As recommended in qualitative analysis, the proposed sample sizes for interviews and focus groups are estimates of the number of individuals needed to reach theoretical saturation of responses. The estimates are based on a sample that includes a range of patient, provider/staff and PRSs perspectives. If theoretical saturation is not met with the proposed sample size, we will seek additional approval to increase our sample size.

### 2.15 Data management and analysis

The research team developed a study-specific data management protocol and standard operating procedures for data collection, quality control, and data extraction. The PI and research coordinators provide ongoing oversight of data management throughout the study and are responsible for checking the accuracy of collected data on an ongoing basis and again prior to data analysis.

All protected health information (PHI) is stored on secure data storage servers or as paper records in a double-locked location at the University of Maryland campus, to which only authorized research personnel have access. Confidentiality is assured as participants are identified on all study materials by only a participant number and date of visit. All data management activities utilize REDCap (Research Electronic Data Capture), a software toolset and workflow methodology for electronic collection and management of research and clinical trial data (53). REDCap provides secure, web-based applications with an intuitive interface for users to enter data and have real time data checking such as validation rules (with automated data type and range checks) at the time of entry. All data input in REDCap are reviewed by a different research assistant to ensure completion and accuracy of data collection. Data files are downloaded from REDCap database on a weekly basis and saved on the study-designated, secure drive to maintain backup copies of the data. De-identified audio recordings and transcripts are stored on secure, password-protected drives at UMCP.

#### 2.15.1 Data analysis

##### 2.15.1.1 Quantitative

Utilizing a conservative intent-to-treat framework, all participants randomized will be included in analyses. We will examine the baseline equivalence of the groups by comparing participants receiving *Peer Activate* and TAU on sociodemographic characteristics, including sex, age, socioeconomic variables, severity of substance use, and days in treatment. Additional preliminary analyses will be conducted, including evaluating distributional assumptions of variables of interest. We will also examine patterns of missingness across treatment groups to detect deviations from missing at random (MAR) assumptions.

Effectiveness outcomes are evaluated primarily at the six-month follow up as well as at the three-month follow up post baseline. Dichotomous outcomes (including retention defined as retained/not retained at each data point) will be examined using generalized linear mixed modeling (GLMM) which allows for the inclusion of both categorical and continuous predictors as well as correlated observations across time. Two-level, mixed-effects models will be used to examine primary hypotheses. Time points (baseline, post-core follow-up, and final follow-up assessments) are nested within participants. Results will be expressed as either odds ratios (for binary, primary outcomes) or mean differences (for continuous, secondary and exploratory outcomes).

Using standard model-building procedures, treatment condition will be entered as the primary predictor in each model. We will then add additional covariates as indicated by our preliminary analyses, including days in treatment and substance use severity. We will also examine a time by group interaction which, if significant, would indicate that differences between treatment conditions become more or less pronounced across assessment points. *Post hoc* differences between treatment condition at three-month and six-month time points will also be examined using a logit link. To model days in treatment, we will conduct survival analysis using a frailty model to predict lapse in treatment and compare survival curves between treatment conditions, including random effect for PRS. We will assess levels of skew in the days in treatment outcome variable and accommodate our approach accordingly.

##### 2.15.1.2 Qualitative and mixed methods

Qualitative interviews and focus groups will be audio-recorded and transcribed for analyses. Analysis of qualitative data will be guided by thematic analysis (54). A team of trained coders will develop detailed codebooks for the patient and staff data (separate codebooks) including definitions and examples for each code. The codebook will be developed iteratively, as done in our prior work. We will use a hybrid deductive-inductive approach for codebook development and analysis. A minimum of two members of the coding team will independently review a set of randomly selected transcripts and identify initial codes using an inductive approach. Coding team members will then discuss their codes and come to a consensus over a final code list of all identified themes grouped based on definitions of implementation outcomes as defined by Proctor’s model (41). Using the shared codebook, coders will code each transcript separately using NVivo and meet to discuss code discrepancies until consensus is reached. The coding team will also inductively identify themes that emerge in subsequent transcriptions and add to the codebook accordingly. We will also conduct a mixed methods analysis, integrating the qualitative feedback on implementation outcomes with quantitative results for each Proctor-defined implementation outcome assessed (41).

### 2.16 Dissemination plan

To disseminate our findings, we will prioritize dissemination to the academic community, PRSs, as well as to the local community. The study site has established two patient research advisory boards, and we will meet regularly with the patient advisory boards to share results from our study and receive input. We will also get their input on how best to disseminate study results to the larger patient population and community.

#### 2.16.1 Dissemination of PRS training

At the end of the trial, if the approach is effective, feasible, and acceptable, we will offer capacity building workshops to train PRSs in the adapted manual, and we also will identify PRS supervisors for training. Feedback from these training sessions will be used to further refine the training and intervention materials and plans for future evaluation and dissemination will be completed. Once completed, this project has the potential to provide immediate benefit to the Maryland community and beyond by making modified training manuals and procedures available for dissemination to other community-based agencies engaging PRSs.

#### 2.16.2 Data sharing plan

We will provide de-identified data from this project following achievement of the aims of the project (i.e., publication of the main outcome paper) through an approved data repository. These data will be provided in digital format (i.e., digital file or access to online repository) with clear labels for all variables. Our team will be available to address queries. All informed consent documents in this study include specific language relating to data sharing and confidentiality and data will also be uploaded to an NIH- and IRB-approved repository per HEAL data sharing requirements.

## 3 Discussion

Evidence-based behavioral interventions to support MOUD outcomes, including methadone, remain limited, including interventions specifically tailored for PRS delivery. Improving methadone retention is essential, and PRSs with their own lived experience with substance use recovery may be uniquely suited to support retention in methadone treatment and address common barriers to methadone retention, particularly among underserved populations. The current hybrid type 1 effectiveness-implementation RCT builds upon our team’s formative work (23, 34, 40, 46) and prior open-label trial to develop and pilot the PRS-delivered BA approach (24). We aim to establish the feasibility, acceptability, and fidelity of *Peer Activate* and effectiveness on improving methadone treatment retention over approximately six months.

### 3.1 Design considerations

We considered recruiting patients initiating methadone treatment or buprenorphine; however, we were concerned that the established differences in retention in methadone treatment vs. buprenorphine would confound our results. We intentionally selected a treatment site that provides both methadone treatment and buprenorphine to expedite the future application of this approach to buprenorphine. However, we also acknowledge that the program given its affiliation with an academic medical center may have greater resources compared to community-based sites without an academic affiliation, including greater support for the peer recovery specialist workforce and other retention supports. Other key considerations relate to the study being a hybrid effectiveness-implementation design (vs. an efficacy trial). We selected TAU as our comparison condition, following recommendations from implementation science to test the incremental clinical benefit of the intervention to existing services, as well as added costs to inform subsequent decisions to adopt the intervention after the trial. In our preliminary work, we have found the greatest challenges with retention in the first month after methadone treatment initiation, and other research also points to the importance of six-month retention for predicting long-term outcomes (55–58). Thus, we elected to focus on six-month methadone treatment retention in this study and designed the trial to lead to a subsequent cluster RCT that includes longer-term outcomes for effectiveness (including one-year retention and opioid abstinence), and implementation outcomes typically assessed well into or after implementation (sustainability, cost effectiveness).

## 4 Conclusion

This trial will test whether *Peer Activate* may be a feasible, scalable, reinforcement-based approach for improving methadone retention. Further, the trial will provide insight as to whether a PRS-delivered BA intervention may be effective and feasible for improving other behavioral health outcomes, such as depression. Positive findings of *Peer Activate* effectiveness may suggest an opportunity to incorporate an evidence-based approach into PRS training nationally.
